# Analysis of laccase‐like enzymes secreted by fungi isolated from a cave in northern Spain

**DOI:** 10.1002/mbo3.1279

**Published:** 2022-03-31

**Authors:** Daniel Fernández‐Remacha, Candela González‐Riancho, Miranda Lastra Osua, Aránzazu González Arce, Itxaso Montánchez, Juan María García‐Lobo, Roger Estrada‐Tejedor, Vladimir R. Kaberdin

**Affiliations:** ^1^ Department of Immunology, Microbiology and Parasitology University of the Basque Country UPV/EHU Leioa Spain; ^2^ IQS School of Engineering Universitat Ramon Llull Barcelona Spain; ^3^ Instituto de Biomedicina y Biotecnología de Cantabria (IBBTEC) CSIC—Universidad de Cantabria Santander Spain; ^4^ Departamento de Biología Molecular Universidad de Cantabria Santander Spain; ^5^ IKERBASQUE, Basque Foundation for Science Bilbao Spain; ^6^ Research Centre for Experimental Marine Biology and Biotechnology (PIE‐UPV/EHU) Plentzia Spain

**Keywords:** *Conidiobolus thromboides*, *Gliomastix murorum*, molecular dynamics simulation, molecular modeling, multicopper oxidase, zymography

## Abstract

Laccases belong to a family of multicopper enzymes able to oxidize a broad spectrum of organic compounds. Despite the well‐known property of laccases to carry out bleaching and degradation of industrial dyes and polyphenolic compounds, their industrial use is often limited by the high cost, low efficiency, or instability of these enzymes. To look for new microorganisms which produce laccases that are potentially suitable for industrial applications, we have isolated several fungal strains from a cave in northern Spain. Their phenotypic analysis on agar plates supplemented with ABTS (2,2′‐azino‐bis(3‐ethylbenzothiazoline‐6‐sulfonic acid)) disclosed two laccase‐positive strains. Further genotyping revealed that they belonged to the *Gliomastix murorum* and *Conidiobolus thromboides* species. The secretion of *G. murorum* and *C. thromboides* laccase‐like enzymes was then confirmed by zymography. Further identification of these polypeptides by mass‐spectroscopy revealed the nature of the laccases and made it possible to predict their functional domains and other features. In addition, plate assays revealed that the laccases secreted by both *G. murorum* and *C. thromboides* were capable of degrading industrial dyes (Congo Red, Indigo, and Eriochrome Black T). Homology modeling and substrate docking predicted the putative structure of the currently uncrystallized *G. murorum* enzyme as well as its amino acid residues potentially involved in interactions with these dyes. In summary, new biochemical and structural insights into decolorization mediated by *G. murorum* laccase as well as identification of laccase‐like oxidase in *C. thromboides* point to a promising future for these enzymes in biotechnology.

## Graphical abstract

This study reports new biochemical and structural insights into decolorization mediated by *Gliomastix murorum* laccase as well as identification of laccase‐like oxidase in *Conidiobolus thromboides*, thus suggesting a promising future for these enzymes in biotechnology.

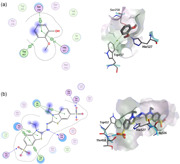

## 1 INTRODUCTION

Reduction‐oxidation (redox) reactions play a central role in several fundamental biological processes (e.g., respiration, energy production, and photosynthesis) evolutionarily conserved across all three kingdoms of life. Consequently, many enzymes that catalyze redox reactions attracted significant interest, in particular oxidases. The latter include numerous laccases (EC 1.10.3.2., *p*‐benzenediol oxidases), a phylogenetically conserved family of enzymes able to oxidize a large variety of organic compounds. The enzymes exert diverse biological functions including copper assimilation in prokaryotes, degradation of decay wood by fungi, and cuticle formation in insects (reviewed in Mate & Alcalde, 2017; Rodriguez Couto & Toca Herrera, 2006).

Owing to the capacity of laccases to carry out catalysis in a wide range of pH and temperature, they are potentially suitable for diverse biotechnological applications. Moreover, given that laccases use molecular oxygen as a co‐factor and produce only water molecules as byproducts, these enzymes are particularly attractive as “green catalysts.”

Among some perspective industrial applications of laccases, lignin degradation in the pulp used for paper production is the best‐known one. Since the whitening of the manufactured paper (due to lignin degradation and bleaching) is usually carried out by employing chlorine‐based compounds, the use of these chemicals can lead to the formation of chlorinated aliphatic and aromatic intermediates, potentially possessing carcinogenic and toxic properties. Although alternative treatment with laccases is more attractive and safer, the absence of inexpensive mediators (necessary to obtain sufficient oxidation rates of nonphenolic lignin structures; Bourbonnais & Paice, 1990) currently limits the use of laccases in paper production.

Degradation of industrial dyes is another well‐known laccase application. A large amount of wastewater containing various industrial dyes and bleaching chemicals is produced daily by the textile industry. These toxic compounds can pollute water with carcinogenic substances such as aromatic amines or azo dyes. Because of their synthetic origin and improved chemical stability, many of these substances do not fade and degrade upon exposure to light or other environmental factors, thus preserving their color and toxicity. The use of laccases could offer a more environmentally friendly way of depolluting wastewater, and some efforts have already been undertaken to test various laccase‐based protocols for dye decolorization (Wesenberg et al., 2003). Other laccase applications include bioremediation of contaminated soil, food processing, and preservation as well as medical care (for a review, see Mate & Alcalde, 2017).

Recent structural studies revealed that pro‐ and eukaryotic laccases belong to a large family of diverse copper‐binding proteins, which contain evolutionarily conserved cupredoxin domains (reviewed in Hakulinen & Rouvinen, 2015). Some cupredoxin domains and/or adjacent sequences frequently include two different copper‐binding sites. The first one (so‐called T1 site) is located in close proximity to the substrate‐binding cavity and is involved in electron transfer to the other trinuclear copper‐binding sites in charge of binding and reduction of O_2_, subsequently resulting in two molecules of water. Several studies demonstrated that both copper‐binding sites are formed by the phylogenetically conserved amino acid residues (i.e., one Cys and nine His residues; Garavaglia et al., 2004) playing along with copper ions an essential role in the laccase‐mediated catalysis. Despite significant progress in understanding the catalytic properties and functions of laccases, still little is known about the structural basis underlying the broad substrate specificities of these enzymes.

Unlike well‐studied microorganisms present in many sites rich in decaying lignocellulosic biomass, bacterial and fungal species originating from unexplored areas could produce laccases with new functions and biochemical properties. This idea prompted us to isolate several fungal species from the caves in northern Spain and screen them for laccase production. Further genotyping of two laccase‐producing fungi revealed that they belonged to the *Gliomastix murorum* and *Conidiobolus thromboides* species. The laccase activity of the corresponding polypeptides was further confirmed by in‐gel assays and the nature of the corresponding laccase polypeptides was determined by mass‐spectroscopy. Moreover, the computer‐assisted analysis of protein structures was used to gain insights concerning the possible contribution of a specific domain and amino acids to the capacity of *G. murorum* laccase to bind and process various organic dyes.

## 2 MATERIALS AND METHODS

### 2.1 Isolation, cultivation, and storage of environmental fungi

A number of fungal isolates were obtained from air samples taken between January and June 2016 in different halls of Altamira Cave (Santillana del Mar, Cantabria, Spain). Two 500 L air samples were simultaneously taken with an air sampler collector SAS‐duo sampler (VWR) for 3 min and directly impinged onto plates with Sabouraud Dextrose Agar (Becton Dickinson) supplemented with 0.5 g/L chloramphenicol (Conda). The plates were transported in an isothermic bag to the lab followed by incubation at 26°C for 3 days.

Several fungal isolates selected for further analysis were cultivated on Potato Dextrose Broth (PDB; Conda) used for the preparation of both liquid and solid (agar) media. To obtain liquid cultures, the isolates were routinely grown in 3 ml of PDB medium supplemented with 30 μg/ml chloramphenicol (Sigma‐Aldrich) at 25°C with shaking at 90 rpm in 12 ml culture tubes. Alternatively, the fungal isolates were grown on PDB agar plates likewise supplemented with chloramphenicol (30 μg/ml). Glycerol stocks were prepared by mixing 450 μl of suspended mycelia and 450 μl of sterile 80% glycerol and were kept at −80°C in 1 ml cryovials. Further inoculations were performed by transferring 20 μl of glycerol stock to solid or liquid PDB media.

### 2.2 Detection of laccases produced by fungal isolates

To test laccase production in liquid cultures, PDB was supplemented with 150 μM of CuSO_4_ and 0.2 mM of ABTS. A dark‐purple color appeared after 5−7 days of incubation and served as an indicator of laccase activity. According to the literature data (Gramms, 2017), the first step of ABTS oxidation leads to the formation of green (cyan) cation radical (ABTS^•+^) predominantly present in the initial phase of this process. However, prolonged incubation with laccases leads to its further oxidation to form a purple product (i.e., ABTS^2+^).

Likewise, the secretion of laccases was also detected on plates by supplementing potato dextrose agar (PDA) with 150 μM of CuSO_4_ and 0.2 mM ABTS (VWR Chemicals) or guaiacol (Merck). Additional assays were carried out in 1.5 ml Eppendorf tubes to confirm the presence of laccase activities in protein preparations used for zymography. These assays were performed by mixing 980 μl of laccase reaction buffer (100 mM sodium acetate buffer, pH 4.5; 0.2 mM ABTS) with 20 μl of protein samples.

### 2.3 Genotypical characterization of fungal isolates

Preparation of genomic DNA was carried out as described by Liu et al. (2000) using 5‐day‐old cultures grown in PDB. The initial identification of each isolate was done by amplification of the genomic D1/D2 region within the large subunit of 28S nuclear ribosomal DNA as well as the Internal Transcribed Spacer (ITS) region (Kwiatkowski et al., 2012). Based on the sequence similarity of the amplified regions revealed by BLAST homology search (https://blast.ncbi.nlm.nih.gov), nearly every isolate was assigned to a particular species (Appendix Table A1). As the above steps were not sufficient to unambiguously identify H18 isolate, its genotyping was completed by using additional primers (see Appendix Table A2) to amplify fragments derived from genes encoding mitochondrial ribosomal RNA of small subunit (mtSSU), nuclear ribosomal RNA of small subunit (SSU), and RNA polymerase II (RPB2). The primer design was carried out with the help of Primer3Plus software available at https://biotools.umassmed.edu/cgi-bin/primer3plus/primer3plus.cgi.

All amplifications were carried out in 50 μl reaction mixtures prepared using Dream Taq PCR Master Mix (ThermoFisher Scientific). The products of amplification were further purified with a GeneJET PCR Purification Kit (ThermoFisher Scientific) and sequenced at the General Genomics Service (SGIker) of the University of the Basque Country UPV/EHU.

### 2.4 Preparation of protein samples

Laccase‐positive fungi were grown in sterile 250 ml Erlenmeyer flasks containing 50 ml of PDB supplemented with chloramphenicol (30 μg/ml) and inoculated with fresh mycelia taken from PDA plates. The flasks were incubated for 4 days at 25°C with agitation (90 rpm) and then production of laccases was induced by adding CuSO_4_ to a final concentration of 150 μM followed by further incubation for 24 h.

The fungal cultures (50 ml each) were transferred from Erlenmeyer flasks to individual 50 ml Falcon tubes and centrifuged for 5 min at 4400*g*. The resulting supernatants (approx. 40 ml) separated from pellets (mycelia and solid particles) were filtered through a Whatman filter paper to remove any remaining parts of the mycelium and other particulates. The filtrates were then passed through a 0.22 μm sterile filter (Millex‐GP PES; Merck Millipore) to remove any remaining cells present in the samples.

The filtrates were stored on ice and further steps were carried out at 4°C. To reduce the volume of the samples, they were concentrated 50 times (i.e., from 15 ml to ca. 300 μl) by transferring them into 15 ml Amicon‐ultra 30 kDa cut‐off filter units (Merck Millipore) followed by centrifugation (20 min, 4400*g*, 4°C). The concentrated sample was separately transferred to 0.5 ml Amicon‐ultra 30 kDa cut‐off filter units (Merck Millipore) and centrifuged for 5 min at 17,000*g* to obtain a final volume of 100−150 μl. Aliquots of each concentrated sample were mixed (4:1) with 5× Laemmli sodium dodecyl sulfate (SDS) sample buffer (ThermoFisher GmbH) and stored at −20°C until further use.

### 2.5 Electrophoretic analysis and zymography

Electrophoretic analysis of extracellular proteins from fungal isolates was performed in standard 15% and 8% acrylamide SDS gels under nonreducing conditions (i.e., in the absence of reducing reagents such as beta‐mercaptoethanol or dithiothreitol) using a Mini‐Protean Tetra Cell (Bio‐Rad Laboratories Inc.) system. The gels used for protein staining were washed in distilled water for 5 min before staining and then were stained with GelCode Blue Safe Protein Stain (ThermoFisher Scientific) for at least 1 h with shaking at 60 rpm. Destaining was done by washing with distilled water, consequently refreshing it every 30 min. To improve the quality of staining, the final destaining step was performed at 4°C overnight.

To carry out activity staining (zymography), gels were equilibrated in 100 mM sodium acetate buffer (pH 4.5) for 10 min, the buffer was replaced with a fresh one, and incubation was repeated for another 10 min. To observe the laccase activity, the gel was overlaid with 5 ml of laccase reaction buffer (2.5 mM ABTS, 100 mM sodium acetate, pH 4.5). The oxidized form (cyan halo) normally became visible during the first 5 min of incubation. To avoid overstaining, the reaction was stopped with 1 mM NaN_3_.

### 2.6 Mass‐spectroscopy identification of proteins

Gel pieces containing the selected proteins were excised manually from gels, cut into smaller pieces, and subjected to in‐gel digestion according to a modified protocol described previously (Shevchenko et al., 2006). Briefly, the excised gel pieces were washed with the solution containing 25 mM ammonium bicarbonate (NH_4_HCO_3_) in methanol/acetonitrile (2:3 v/v); proteins were reduced with 10 mM dithiothreitol (DTT)/50 mM NH_4_HCO_3_, alkylated with 25 mM iodoacetamide/50 mM NH_4_HCO_3_ at room temperature for 30 min, washed with molecular biology grade water and vacuum dried. Further trypsin digestion was performed with trypsin solution (12.5 ng/μl trypsin; Roche Diagnostics in 25 mM ammonium bicarbonate containing 10% (v/v) acetonitrile) for 12−16 h at 37°C. The supernatant was recovered and peptides were extracted twice from the gel: first, with 25 mM NH_4_HCO_3_ and acetonitrile and then with 0.1% trifluoroacetic acid and acetonitrile. The recovered supernatants and the extracted peptides were pooled, dried in a SpeedVac (ThermoFisher Scientific), and subsequently desalted with homemade C18 tips (3M Empore C18).

Mass spectrometric (MS) analyses were performed on an EASY‐nLC 1200 liquid chromatography system interfaced with a Q Exactive HF‐X mass spectrometer (ThermoFisher Scientific) via a nanospray flex ion source. Desalted peptides were loaded onto an Acclaim PepMap100 precolumn (75 µm × 2 cm; ThermoFisher Scientific) connected to an Acclaim PepMap RSLC (75 µm × 25 cm; ThermoFisher Scientific) analytical column. Peptides were eluted from the column using the following gradient: 18 min from 2.4% to 24%, 2 min from 24% to 32%, and 12 min at 80% of acetonitrile in 0.1% formic acid at a flow rate of 300 nl/min. The mass spectrometer was operated in positive ion mode. Full MS scans were acquired from *m/z* 375 to 1850 at a resolution of 120,000 (*m/z* 200). The 10 most intense ions were fragmented by higher energy C‐trap dissociation with a normalized collision energy of 28 and MS/MS spectra were recorded with a resolution of 15,000 (*m/z* 200). The maximum ion injection time was 100 ms for survey scans and 120 for MS/MS scans, whereas AGC (Automatic Gain Control) target values of 3 × 10^6^ and 5 × 10^5^ were used for survey and MS/MS scans, respectively. To avoid repeated sequencing of peptides, dynamic exclusion was applied for 12 s. Singly charged ions or ions with unassigned charge states were also excluded from MS/MS. Data were acquired using Xcalibur software (ThermoFisher Scientific).

Raw files were processed with Proteome Discoverer 2.2 (ThermoFisher Scientific) against a UniProtKB database. Precursor and fragment mass tolerances were set to 10 ppm and 0.02 Da, respectively, and up to two missed cleavages were allowed. Carbamidomethylation of Cys was set as fixed modification and oxidation of Met and protein N‐terminal acetylation as variable modifications. Protein Discoverer generated peak list was also used for the de novo peptide sequencing using PEAKS Studio 5.3 (Bioinformatics Solutions Inc.). Precursor and fragment mass tolerances were set to 10 ppm and 0.02 Da, respectively. Carbamidomethylation of Cys was set as fixed modification and oxidation of Met as variable modification. Results were filtered for TLC > 3 (total local confidence) and ALC > 30 (average local confidence) and resulting peptide lists were exported as CSV files for further analysis.

### 2.7 Degradation of industrial dyes

PDA plates supplemented with chloramphenicol (30 μg/ml) and 0.2 g/L dye (i.e., Congo Red, Indigo carmine, or Eriochrome Black T all supplied by Merck) were inoculated with mycelium placed in the center of each plate. The plates were incubated at room temperature for 5 days and then examined for the presence of halos corresponding to the areas, in which the dyes were degraded.

To carry out dye decolorization assays in liquid cultures, the latter were prepared by transferring 100 μl glycerol stock of *G. murorum* to 3 ml of YEPD (yeast extract peptone dextrose) (Merck) medium followed by incubation at 25°C with shaking (200 rpm) for approximately 3 days and then dyes (i.e., Congo Red or Eriochrome Black T) were added. Both *G. murorum* inoculates and 3 ml aliquots of YEPD medium (controls) containing either dye at a concentration of 0.2 g/L (or 0.1 g/L) were incubated under the same conditions (25°C, 200 rpm) for 4 days. The optical densities of YEPD medium (background) and aliquots of the control and test samples withdrawn after 1 and 4 days of incubation were recorded at 635 nm (OD_635_) using a 96‐flat‐wells plate (ThermoFisher Scientific) and Synergy™ HT Multi‐Detection Microplate Reader (BioTek®; Agilent). While calculating the percentages of the dyes remaining after incubation of test samples, the optical density of the corresponding controls (after deduction of background) was considered as 100%. All decolorization assays were carried out in the absence of mediators.

### 2.8 Testing the temperature‐dependence of *G. murorum* laccase

To test the dependence of *G. murorum* laccase activity on temperature, enzymatic assays were performed in a 96‐well flat‐wells plate (ThermoFisher Scientific) whose wells were filled with 398 μl of laccase reaction buffer (100 mM sodium acetate buffer, pH 4.5; 0.2 mM ABTS) used in triplicate for the test (enzymatic assays) and control (buffer along) samples. Once the plate was preheated in the plate‐reader (Synergy™ HT Multi‐Detection Microplate Reader; BioTek®; Agilent) to 20°C, 30°C, 40°C or 50°C, 2 μl of *G. murorum* protein sample (prepared as samples used for zymography) was added to the test samples and the absorbance of all samples was read for 20 min with 1 min intervals.

### 2.9 Homology modeling and substrate docking

The *G. murorum* laccase model was inferred using MOE2019.01 (ULC CCG, 2021). The sequence of *Acremonium murorum* laccase was retrieved from Uniprot (ID: Q9P8C3), and templates were searched based on sequence similarity using BLAST. The best match with 66% of identity and 88% of query coverage was selected as a reference. The corresponding structure; that is, the *Albifimbria verrucaria* bilirubin oxidase, was then retrieved from the Protein Data Bank (PDB ID: 6IQZ) and further used for homology modeling. The full‐length protein was modeled, including the signal peptide. The latter was generated with the MOE loop modeler due to its absence in the template structure. GB/VI scoring function and 0.5 Å RMS gradient limit was set to score the generated models. The best model was superposed with the template to include the copper atoms and optimize the orientation of the copper interacting residues in the mononuclear and trinuclear sites. The system was energy minimized while progressively reducing the positional restraints (starting at 20 kcal/mol/Å^2^) on the multicopper site, which includes copper atoms and coordinating histidine and cysteine residues. The resulting model was then further processed using the tLEaP module included in the Amber20 Molecular Dynamics (MD) software (Case et al., 2021) considering the AMBER*ff*14SB force field (Maier et al., 2015). The system was neutralized and solvated with the OPC water model (Izadi et al., 2014) defining a truncated octahedron (10 Å radius) before proceeding to MD simulations. Simulations were computed with the SANDER and PMEMD modules using the SHAKE algorithm to constrain all bonds involving hydrogens at all stages and employ an 8 Å cutoff for nonbonded interactions. The system was initially energy minimized in two stages: first, a 5000 steps minimization using Steepest Descent method (switched to conjugate gradient after 1500 steps) was done, applying a 20 kcal/mol/Å^2^ restraint to the protein, followed by a second minimization maintaining only the restraints on the multicopper site.

The system was then heated during 0.5 ns to 300 K using the Langevin thermostat in a canonical NVT collective and 0.002 ps time step. A density stage was carried out in NPT conditions to allow stabilization of the density of the system, followed by a preproduction stage (0.5 ns each) to let the whole system equilibrate. Finally, the positional restraint on the copper‐binding site was reduced to 10 kcal/mol/Å^2^ and a 500 ns production stage was conducted under the NPT collective. Such positional restraint was necessary to maintain the copper coordinating interactions while avoiding excessive biasing of the system. The resulting trajectory was analyzed using cpptraj (Roe & Cheatham, 2013) to perform a basic analysis of the system, including RMSD, RMSF, and principal components analysis. The trajectory was also clustered using the DBScan method (Ester et al., 1996). The best performance, in this case, was achieved by setting the minimum number of points, which were sufficient to form a dense region (minpoints), to 4 and *ε* = 1.18. The method was optimized as recommended in the AMBER manual (Case et al., 2021) by first generating a “K‐Dist” plot to select the minpoints value and then performing several runs while varying *ε* (in our case *ε* ranged from 1 to 1.2 with increments of 0.02). The best cluster result was selected following a combined criterion of highest pseudo‐*F* statistic (pSF) and lowest Davies−Bouldin Index (DBI) values (Shao et al., 2007), which were further examined to avoid the presence of singletons and equally sized clusters (which tend to give low DBI and higher pSF values, respectively). The dominant cluster was used to assess the binding mechanisms of considered laccase substrates by molecular docking.

MOE software was used to perform molecular docking. The AMBER14:EHT force field was considered to adequately prepare all molecular systems. Several protonation states were tested (going from pH 4 to 7), and no differences were observed in the dominant protonation state. Therefore, the molecules were energy minimized and used for docking calculations. Following the homology model approach, the same ligand binding site as in *A. verrucaria* bilirubin oxidase was assumed to be present in the generated model. Consistently, the MOE site finder utility also detected this area as a possible ligand‐binding site. Thus, the studied compounds were docked within this site of the protein (i.e., in close proximity to His527 coordinating the T1 copper ion involved in the electron transfer) using induced fit conditions and a 6 Å cutoff. London dG and GBVI/WSA dG scoring functions were used for the placement and refinement, respectively. The best conformation for each ligand was selected for studying the binding mechanism.

## 3 RESULTS

### 3.1 Isolation and genotyping of fungi

While searching for fungal species potentially producing new laccases, we have analyzed 20 fungal isolates recently obtained from air samples collected in the Cave of Altamira. Rapid screening for laccase production in liquid cultures by using ABTS as a substrate made it possible to preselect six putative laccase‐positive strains. To identify these fungal isolates, we determined the sequences of their ITS and D1/D2 regions and did a BLAST run against the sequences available in the NCBI database (for details, see Section 2). Based on the BLAST results (see Appendix Table A1), only H2 and H13 were unambiguously identified as *Epicoccum nigrum* and *G. murorum* species, respectively. To complete the identification of other isolates, we employ a multi‐*locus* sequence analysis (MLSA). Namely, the genomic DNA of these isolates was amplified with additional pairs of primers (see Appendix Table A2) and the sequence of the corresponding amplicons was used for BLAST search to identify the best matches. The summary of this analysis is presented in Table 1, indicating that the remaining isolates (H4, H8, H10, and H18) belonged to *E. nigrum* (H4 and H8), *Fusarium solani* (H10), and *Coniodiobolus thromboides* (H18) species.

**Table 1:** Genotyping of fungal isolates

Strain ID	Gene	Species	Coverage of the query (%)	Homology (%)
H2	*FU1*	*Epicoccum nigrum*	100	100
	*FU2*	*Epicoccum nigrum*	99	99
H4	*FU1*	*Epicoccum nigrum*	100	99
	*FU2*	*Psiloglonium pusillum*	100	99
	*TEF1*	*Epicoccum sp.*	99	100
	*RPB2*	*Epicoccum poae*	100	95
	*TUB2*	*Epicoccum nigrum*	58	99
H8	*FU1*	*Epicoccum nigrum*	100	100
	*FU2*	*Phoma* sp.	100	100
	*TUB2*	*Epicoccum nigrum*	99	99
H10	*FU1*	*Fusarium solani*	100	99
	*FU2*	*Fusarium solani*	100	99
H13	*FU1*	*Gliomastix murorum*	100	98
	*FU2*	*Gliomastix murorum*	100	99
H18	*FU1*	*Malassezia restricta*	97	99
	*FU2*	*Conidiobolus. thromboides*	100	100
	*mtSSU*	*Conidiobolus thromboides*	100	100
	*SSU1*	*Conidiobolus thromboides*	97	99
	*SSU2*	*Conidiobolus thromboides*	100	99

*Note*: Genomic DNAs extracted from fungal isolates H2, H4, H8, H10, H13, and H18 were PCR‐amplified with several locus‐specific primers listed in Appendix Table A2 and the resulting amplicons were sequenced. Based on the sequence homology (≥99%) revealed by BLAST (https://blast.ncbi.nlm.nih.gov/Blast.cgi) search in the NCBI database, the isolates were assigned to *Epicoccum nigrum* (H2, H4, and H8), *Fusarium solani* (H10), *Gliomastix murorum* (H13), and *Coniodiobolus thromboides* (H18).

Abbreviation: PCR, polymerase chain reaction.

### 3.2 Detection of laccase production by environmental isolates

**Figure 1:**
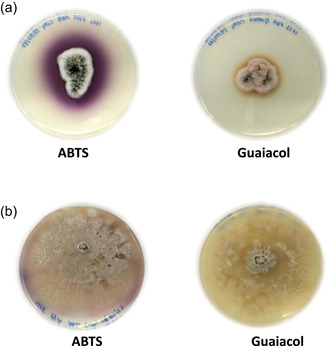
Detection of laccases secreted by environmental isolates of *Gliomastix murorum* (a) and *Conidiobolus* *thromboides* (b). Assays were performed on potato broth plates supplemented with ABTS or guaiacol. ABTS, 2,2′‐azino‐bis(3‐ethylbenzothiazoline‐6‐sulfonic acid

**Figure B1:**
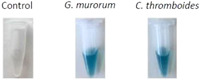
Laccase activity detection assay performed with*Penicillium commune*, *Gliomastix murorum*, and *Conidiobolus thromboides* samples. Detection of laccase activity was carried out in Eppendorf tubes according to the procedures described in Section 2. Incubation of reaction buffer alone (control) is also shown

To validate laccase secretion, each isolate was grown on PDA plates supplemented with ABTS or guaiacol. These experiments revealed that only two isolates identified as *G. murorum* and *C. thromboides* were able to secrete laccase‐like enzymes, respectively forming purple or orange halos around the fungal mycelia (Figure 1). In addition, in vitro assays likewise confirmed the presence of extracellular laccases secreted by these fungi grown in a liquid medium (Appendix Figure B1).

**Figure 2:**
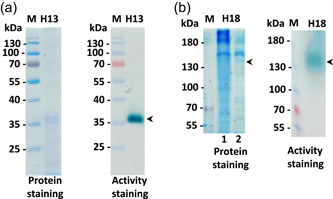
Detection of *Gliomastix murorum* and *Conidiobolus thromboides* laccases by zymography. Aliquots (15 μl) of protein samples containing polypeptides (approx. 200−500 ng) secreted by *G. murorum* (panel a, lane H13) or *C. thromboides* (panel b, lane H18) polypeptides along with prestained protein marker (PageRuler™; Fisher Scientific) were subjected to electrophoretic fractionation at 200 V in 12% or 8% polyacrylamide gels, respectively. One part of the gel was stained with GelCode Blue Safe Protein stain (protein staining), whereas the other part was used for zymographic detection of laccases (activity staining). Arrowheads indicate the positions of polypeptides that show laccase activity. Lines 1 and 2 in the gel used for protein staining (panel b, on the left) correspond to undiluted and diluted (1:2) *C. thromboides* protein samples, respectively

Once confirmed, the secretion of laccases by *G. murorum* and *C. thromboides* was further analyzed by zymography to identify polypeptides in charge of these activities. Namely, aliquots of cultures were separated from mycelia by centrifugation and the supernatants containing polypeptides secreted by *G. murorum* and *C. thromboides* were sequentially filtered, concentrated, and mixed with nonreducing protein loading buffer. The resulting samples of partly purified proteins from each species were separated on SDS polyacrylamide gels. Analysis of *G. murorum* proteins revealed that the polypeptide that possesses a laccase‐like activity was migrating as an approx. 37 kDa protein (Figure 2a). A similar analysis performed for extracellular proteins secreted by *C. thromboides* demonstrated that the corresponding laccase activity was associated with a polypeptide(s) with a molecular mass close to 130 kDa. The polypeptides corresponding to the putative *G. murorum* and *C. thromboides* activities were further identified by mass‐spectroscopy. Analysis of mass‐spec data obtained for the *G. murorum* revealed that they fully match the sequence of the laccase polypeptide with the predicted molecular weight of 66.92 kDa and accession number CAB75422.1 in the NCBI database. The faster mobility of this polypeptide in protein gels was likely due to semi‐denaturing conditions (i.e., the absence of reducing reagents and lack of sample preheating prior electrophoresis) that we used to facilitate the recovery of enzymatic activities in zymographic assays after electrophoresis.

**Figure 3:**
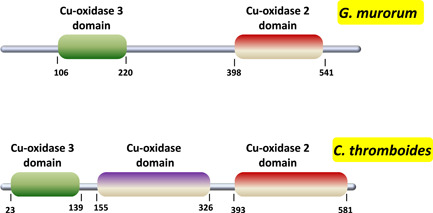
Schematic structures and functional domains of *Gliomastix murorum*and *Conidiobolus thromboides* laccases. The search for Pfam functional domains was carried out by using online tools available at http://pfam.xfam.org/. This analysis revealed the presence of three cupredoxin‐like domains in the *G. murorum* and *C. thromboides* laccases, respectively. Moreover, both proteins also contain signal peptides (amino acids 1−21 and 1−15, respectively)

Further sequence analysis of this polypeptide at https://www.ncbi.nlm.nih.gov/Structure/cdd/wrpsb.cgi revealed that it is composed of three cupredoxin domains (see Figure 3) phylogenetically conserved in many fungal laccases. Moreover, according to the information provided for this polypeptide in UniProt at https://www.uniprot.org/uniprot/Q9P8C3, it also possesses a signal peptide (residues 1−21). As to peptides present in the trypsin digest of *C. thromboides* fraction, advanced in silico analysis was performed by running a BLAST search to find a match among the entries of the Integrated Microbial Genomes & Microbiomes system (IMG/M: https://img.jgi.doe.gov; Chen et al., 2021) containing the partly annotated genome of *C. thromboides* FSU 785. This analysis revealed a hypothetical polypeptide (Locus Tag 342241) composed of three cupredoxin domains (see Figure 3) with a signal peptide (residues 1−15) at its N terminus.

### 3.3 Assessing the capacity of *G. murorum* and *C. thromboides* laccases to decolorize industrial dyes

**Figure 4:**
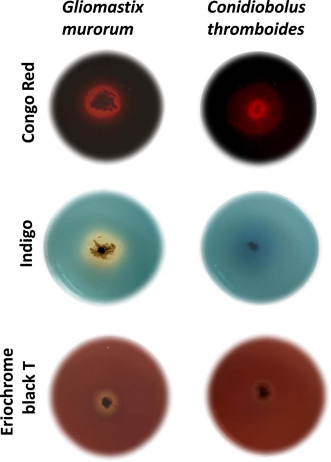
Phenotypic assays assessing the capacity of *Gliomastix* *murorum* and *Conidiobolus* *thromboides* to degrade industrial dyes. Fresh mycelia of *G. murorum* and *C. thromboides* were used to inoculate PDA plates supplemented with 0.2 g/L industrial dyes (Congo Red, Indigo, or Eriochrome Black T), and further incubation was carried out at 20°C for 5 days. The putative laccase activities were revealed by the appearance of the characteristic halos formed around the growing mycelia

**Figure 5:**
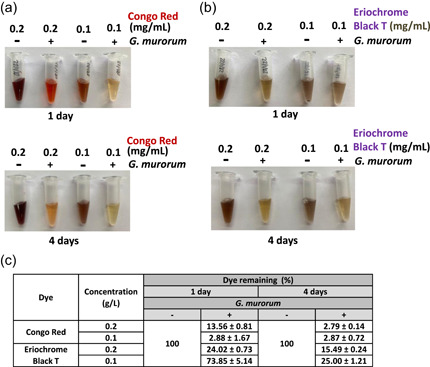
Decolorization assays. Congo Red or Eriochrome Black T were added to fresh cultures obtained by inoculation of YEPD medium with *Gliomastix murorum* (test cultures) or to YEPD medium alone (control) at concentrations of 0.2 and 0.1 mg/ml, respectively, and the cultures were further incubated as described in Section 2. Aliquots of mycelium‐free test and control samples were withdrawn after 1 and 4 days of incubation (panels a and b, respectively), and their absorbance, as well as the absorbance of YEPD medium (background), was recorded in triplicate to calculate the percentage of the remaining dyes (panel c). YEPD, yeast extract peptone dextrose

**Figure B2:**
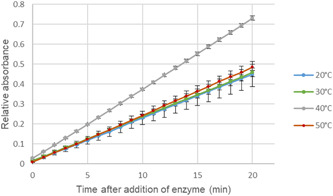
Temperature‐dependent oxidation of ABTS by *Gliomastix* *murorum* laccase. The assays were performed using a 96‐well plate at 20−50°C and the absorbance was recorded at 409 nm during the first 20 min after the addition of the partially purified enzyme. The relative absorbance corresponds to mean values obtained in three independent measurements and the standard deviations are indicated by vertical bars. The highest activity was observed at 40°C. ABTS, 2,2′‐azino‐bis(3‐ethylbenzothiazoline‐6‐sulfonic acid

As one of the potential biotechnological implications of fungal laccases is decolorization of industrial dyes, the susceptibility of several dyes to oxidation by *G. murorum* and *C. thromboides* laccases was also assayed. Experiments were performed with both laccase‐positive fungi individually grown on agar plates containing either Congo Red, Indigo carmine, or Eriochrome Black T. The formation of decolorization zones (halos) formed around the growing mycelia served as an indication of dye oxidation by the respective secreted enzymes. As seen in Figure 4, the fungal isolates possess different capacities to oxidize the aforementioned dyes. Namely, in contrast to the lack of halos or their lower intensities on plates inoculated with *C. thromboides*, the halos produced by *G. murorum* looked rather intense, especially in the case of plates with Congo Red and Eriochrome Black T. The decolorization of these dyes by *G. murorum* was further tested by using liquid cultures and different concentrations of dyes (see Figure 5). Analysis of these cultures revealed (see Figure 5c) that efficient degradation has already occurred after 1 day (i.e., the percentage of remaining dyes was ca. 13% and 24% for concentrated [0.2 g/L] solutions of Congo Red and Eriochrome Black T, respectively) and incubation for 4 days could nearly completely decolorize Conco Red and reduced the concentration of Eriochrome T by fourfold. Additional in vitro assays carried out at 20°C, 30°C, 40°C, and 50°C using ABTS as a substrate revealed that the optimal oxidation rate by *G. murorum* laccase was observed at 40°C (Appendix Figure B2).

### 3.4 Computer modeling of *G*. *murorum* laccase and its interaction with substrates

The capacity of *G*. *murorum* laccase to decolorize/oxidize several industrial dyes prompted us to gain further insights underlying the structure and substrate‐binding properties of this enzyme. As the experimentally determined structure of this enzyme is not available, we decided to predict it by homology modeling. While searching for a template, we found that, except for *A. verrucaria* bilirubin oxidase, other fungal copper oxidases that possess known three‐dimensional structures showed a low percentage of sequence similarity (see Appendix Table A3). Since the sequence of *A. verrucaria* bilirubin oxidase was remarkably similar to that of *G. murorum* and the *A. verrucaria* homolog likewise possessed a laccase‐like activity, this protein was employed as a scaffold to model *G. murorum* laccase.

**Figure 6:**
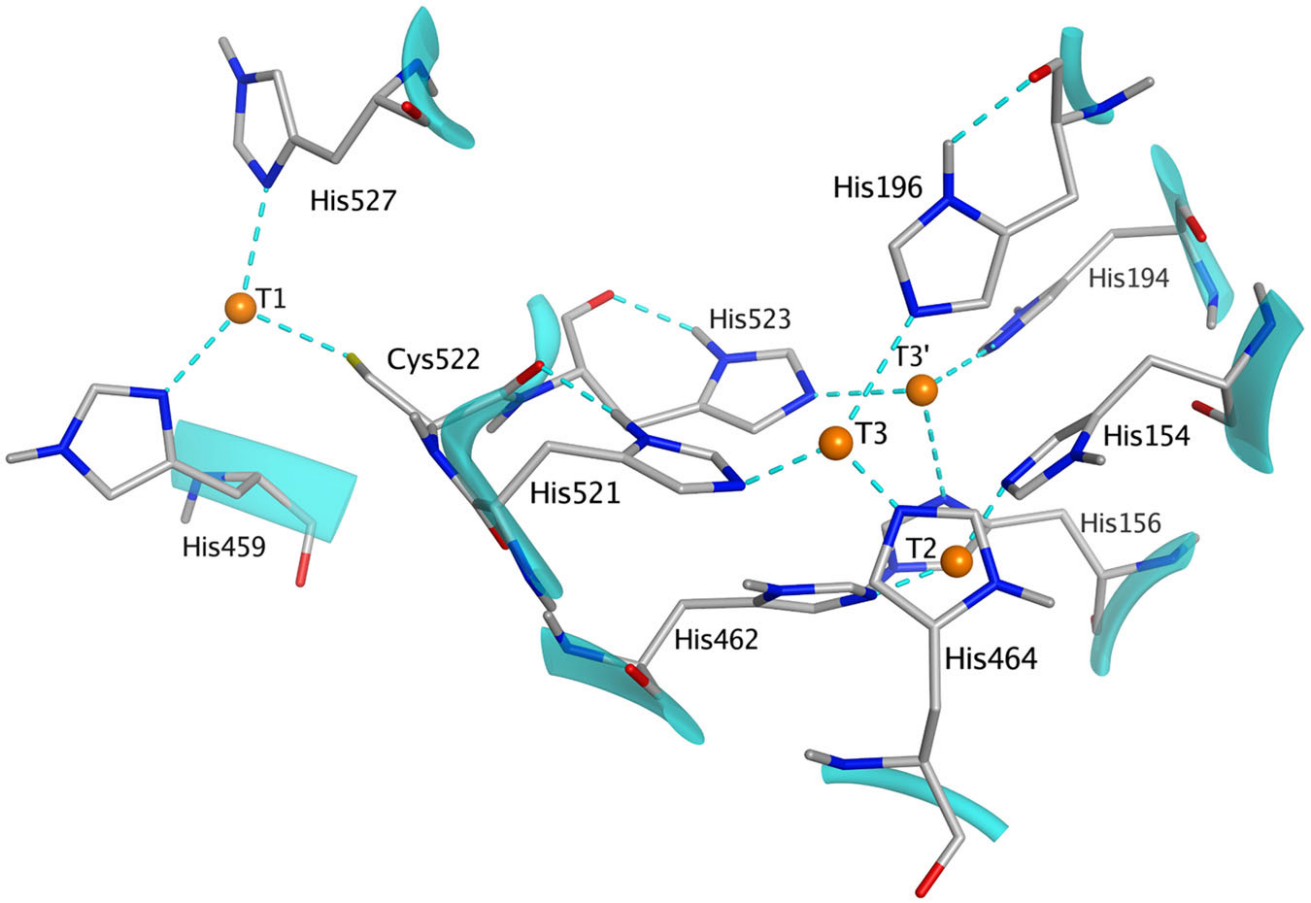
Interactions of superposed copper atoms with Cys and His residues residing in the mononuclear and trinuclear sites. The interactions shown by dashed lines were deduced after geometry optimization of the surrounding residues

The obtained homology model was superimposed onto its template to add the putative copper ions. As seen in Figure 6, the predicted location of the copper ions and their interactions with the neighboring amino acid residues is in agreement with the interactions described by Hakulinen and Rouvinen (2015). In the trinuclear site, both T3 copper atoms interact with adjacent histidines and the T2 copper ion is coordinated by two other histidines, thus anticipating the involvement of eight distinct histidine residues. Concerning the mononuclear site, the T1 copper atom is coordinated by two histidines and one cysteine residue. Therefore, the coordination network in the multinuclear site is consistent with that reported by others (Bertrand et al., 2002; Kallio et al., 2009; Matera et al., 2008). Following the addition of copper ions, the full system was solvated and prepared to perform 500 ns long MD simulations.

**Figure 7:**
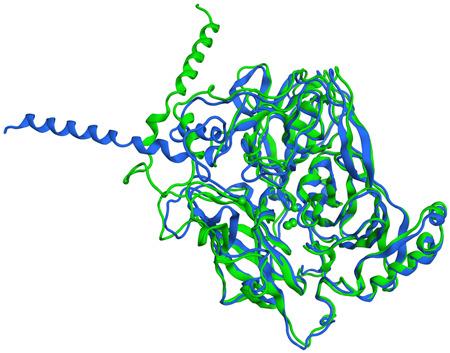
Superimposition of structures representing Cluster 0 (green) and Cluster 1 (blue) including copper atoms shown as spheres with Van der Waals radius

**Figure B3:**
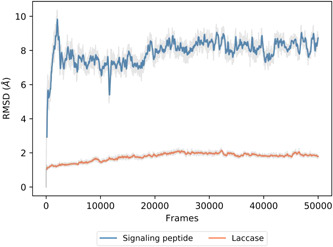
Moving average over the RMSD of the signaling peptide (blue) and the rest of the laccase (orange) during the molecular dynamics simulation. The gray shadows behind each series show the original data

**Figure B4:**
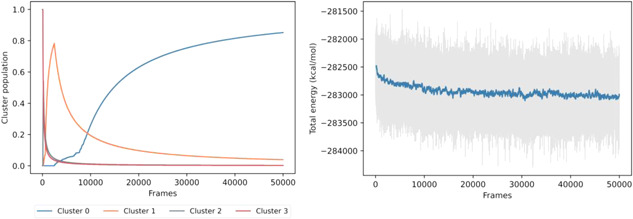
Dynamics of cluster populations (on the left) and total energy (on the right) observed during the simulation. (Left panel) Cluster population versus time. The DBScan algorithm was applied to the backbone of the protein to perform the clustering analysis. (Right panel) The running average (using a window of 150 points) of the total energy of the system (kcal/mol) is represented by a blue curve, whereas the corresponding raw data are in light gray

**Figure B5:**
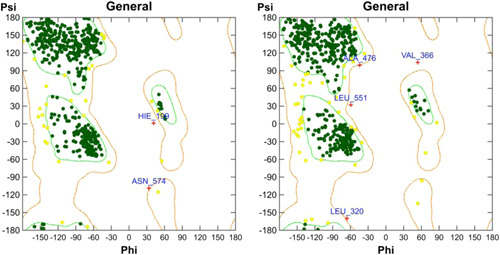
Ramachandran (Phi−Psi) plots of Cluster 0 (on the left) and Cluster 1 (on the right). Residues with outlying *phi−psi* angles are indicated with red crosses

The stability of the system was assessed by monitoring the RMSD and energy values along the simulation. Clustering analysis was also performed to find the most representative structure produced during the MD simulation. RMSD analysis of the MD showed a progressive evolution of the system during the first 250 ns (25,000 frames), where a noticeable increment can be seen (Appendix Figure B3), indicative of a substantial conformational change in the geometry of the protein backbone. The rest of the simulation showed a rather constant RMSD profile, thus indicating that the system evolved toward a more stable conformation. This fact is further corroborated by cluster analysis. The latter yielded four clusters (Appendix Figure B4), whose profiles demonstrated a fast transition of the system toward a lower energy conformation due to the fast decrease of Clusters 1, 2, and 3, and the progressive increase of Cluster 0. Consistently, the total energy of the system decreases at the beginning and rapidly reaches the equilibrium phase indicating a transition toward a more structured and stable conformation. The difference between the two main clusters is primarily manifested by the position of the signaling peptide, which is more exposed to the solvent in Cluster 1 (blue). This conformation is less stable than the one seen in Cluster 0 (green), in which the signaling peptide closely interacts with the rest of the protein (Figure 7) and has a higher number of residues in the core regions of the Ramachandran plot (Appendix Figure B5). Both clusters show adequate backbone structures with very few outliers (Appendix Figure B5), and none of the outlying residues is involved in copper binding or electron transfer.

The representative structure obtained from Cluster 0 was used to perform docking analysis and to study protein−ligand interactions. Similar to the location of the substrate‐binding site (SBS) in other laccases (see by Kallio et al., 2009), the putative SBS of *G. murorum* laccase appears to be buried between the first and second cupredoxin domains, and its shape is likely delimited by five protein loops derived from these domains. The model also predicts that the SBS site contains the T1 coordinating histidine exposed to the solvent, and therefore this residue could be accessible for interaction with substrates. Moreover, the substrate‐binding pocket looks rather lipophilic because of the presence of two tryptophan (i.e., Trp422 and Trp457) and two isoleucine (i.e., Ile364 and Ile526) residues. Their presence in a deep cavity likely facilitates interaction with π‐electron‐rich motifs such as those present in conjugated or aromatic compounds.

**Figure 8:**
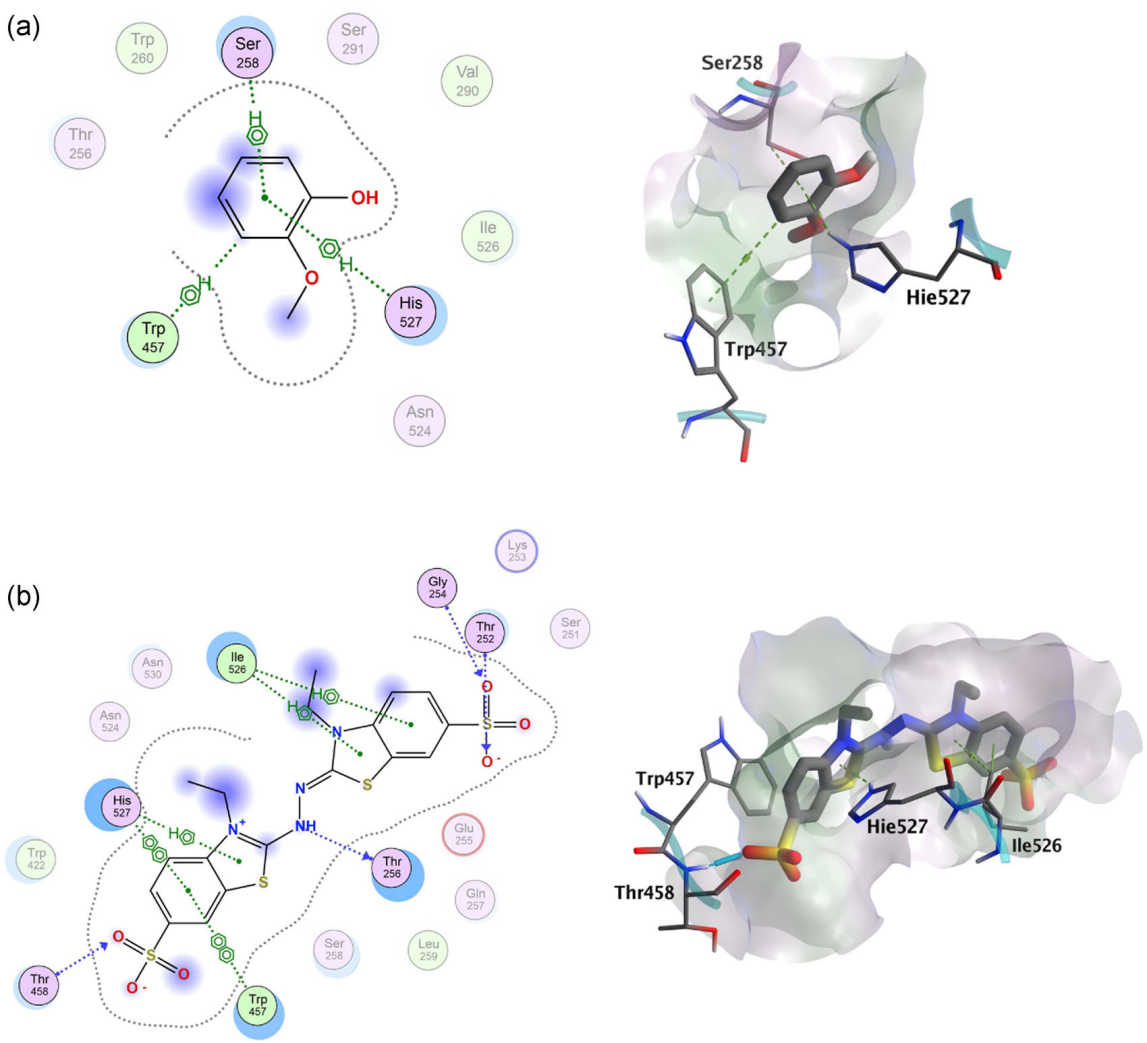
Binding modes of guaiacol (a) and ABTS (b) to *Gliomastix* *murorum* laccase. Noninteracting residues are shadowed in two‐dimensional depictions and only those residues near His527 are shown in the three‐dimensional representation. ABTS, 2,2′‐azino‐bis(3‐ethylbenzothiazoline‐6‐sulfonic acid

**Figure B6:**
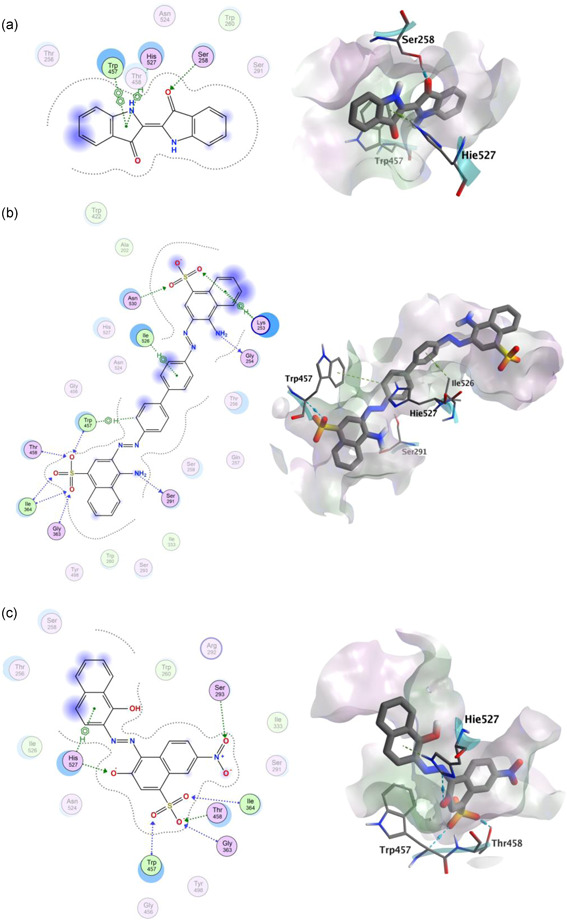
Binding modes of Indigo (a), Congo Red (b), and Eriochrome Black T (c) to *Gliomastix* *murorum* laccase. Noninteracting residues are shadowed in two‐dimensional depictions and only those residues near His527 are shown in the three‐dimensional representation

Once the model of the *G. murorum* laccase was completed, we further explored it for its interactions with a variety of substrates. Namely, we used molecular docking to reveal the contacts determining the interactions of the enzyme with ABTS and several dyes tested in decolorization assays shown in Figure 4. The ligand‐binding site of docked structures was analyzed to study changes induced by the compounds on the pocket of the laccase model. Such changes turned out to be very small with an average RMSD value of 0.07 ± 0.02 Å. In addition, a number of amino acid residues potentially contributing to the substrate‐binding were identified (Figure 8 and Appendix Figure B6).

Docking results agree with the previously published data that some ligands indirectly interact with the T1 copper atom. Namely, this interaction is mediated by a histidine residue coordinated by the T1 copper ion (Hakulinen & Rouvinen, 2015). The corresponding histidine residue (i.e., His527) in the *G. murorum* laccase is equivalent to His458 in *Trametes versicolor* (Bertrand et al., 2002), His431 in *Melanocarpus albomyces* (Kallio et al., 2009), and His455 in *Trametes trogii* (Matera et al., 2008) oxidases and, similar to its counterparts, it appears to serve as a primary electron acceptor in the electron transfer chain. Our results revealed that all ligands, except Congo Red, can interact with His527 via hydrogen−π interactions. The commonality of other enzyme−ligand interactions is generally more difficult to infer for laccases known for their ability to oxidize a wide variety of structurally unrelated compounds (Jones & Solomon, 2015). In this respect, other less conserved residues, such as Trp457, and Thr458 as well as some serine residues were predicted to also interact with the docked molecules, thus suggesting their involvement in binding laccase substrates.

## 4 DISCUSSION

Microorganisms serve as an important source of industrial enzymes widely used in diverse biotechnological processes including biofuel production, bioremediation, and biochemical synthesis of rare compounds. While oxidases, in particular laccases, have demonstrated valuable characteristics of “green enzymes,” their use in the industry run into several limitations (low stability, high cost of enzymes and redox mediators, or lack of specificity) that still hinder the wide use of these enzymes (Arregui et al., 2019).

To expand the number of perspective laccases potentially suitable for industrial applications and characterize their properties, we have obtained several fungal isolates from the cave of Altamira. Their preliminary screening on selective media commonly used to detect laccase secreting organisms made it possible to identify two laccase‐producing strains further genotyped by using a number of fungi‐specific primers. The MLSA revealed that the laccase‐positive isolates belonged to the *G. murorum* and *C. thromboides* species and therefore were subsequently named *G. murorum* CA1 and *C. thromboides* CA1. While the existence of the *G. murorum* laccases was previously envisaged based on the experimental data (Gouka et al., 2001), we report here the laccase activity of *Conidiobolus* species. The presence of laccase‐like activities was corroborated by in vitro assays using ABTS as a substrate (see Appendix Figure B1). These experiments revealed that *G. murorum* CA1 migrates as a 37 kDa polypeptide, which was further digested with trypsin and analyzed by mass‐spectroscopy. The mass‐spec data confirmed that the polypeptide secreted by *G. murorum* CA1 is nearly identical to the previously annotated laccase of *A. murorum* (see Mehra et al., 2018). Unlike *G. murorum* CA1 laccase, the *C. thromboides* CA1 polypeptide with laccase activity was migrating as a 130 kDa protein under semi‐denaturing conditions. The relatively high molecular weight of this polypeptide(s) is likely indicative of the dimeric/trimeric nature of the enzyme, which is consistent with the propensity of some fungal laccases to function as dimer/trimers (Hakulinen & Rouvinen, 2015).

While testing the capacity of both laccases to decolorize industrial dyes, we found that *G. murorum* enzyme was able to efficiently oxidize Congo Red, Indigo, and Eriochrome Black T (Figure 4), whereas plate assays carried out with other isolates revealed the low reactivity of *C. thromboides* enzyme toward these dyes. The capacity of *G. murorum* to efficiently decolorize Congo Red and Eriochrome Black T was further corroborated by the results of decolorization assays carried out in liquid cultures (Figure 5), thus reinforcing the idea that the *G. murorum* oxidase might be a promising enzyme for future biotechnological applications. Moreover, further in vitro analysis of *G. murorum* laccase activity using ABTS revealed that the maximal oxidation rate of this substrate occurred at 40°C (Appendix Figure B2).

To better understand the structural features underlying the organization of *G. murorum* laccase and its capacity to oxidize industrial dyes, we next modeled its three‐dimensional structure using *A. verrucaria* bilirubin oxidase as a template. The high sequence similarity of both enzymes suggests that the *G. murorum* homolog might belong to a subclass of oxidases closely related to fungal laccases. The initial inspection of the homology model indicated that the model maintained the overall architecture of laccase‐like enzymes including three well‐structured cupredoxin domains. Moreover, the orientation of the conserved His residues closely resembled that of other laccases (e.g., laccases of *M. albomyces* and *T. versicolor*). Besides the core structure, the model also included the signal peptide (amino acids 1−21), a relatively conserved helix structure exhibiting higher mobility than the rest of the protein. As a result, the main difference between two major conformations representing Cluster 0 and Cluster 1 was in the position of this helix (Figure 7).

Although the biochemical properties of some laccases are well studied, much less is known about the structural basis determining their interaction with various substrates. Recent attempts to characterize the binding modes of *T. versicolor* and *Cerrena versicolor* laccases made it possible to confirm the crucial role of some amino acid residues (e.g., Asp206 and His458, *T. versicolor* numbering) in assisting substrate binding (Mehra et al., 2018). However, owing to the very low sequence similarity (ca. 18%) between *T. versicolor* and putative *G. murorum* oxidases (see Appendix Table A3), the structural features used to explain substrate binding by *T. versicolor* laccase unlikely resemble those of *G. murorum*. These considerations prompted us to use molecular docking to reveal the nature of contacts potentially employed by the *G. murorum* enzyme to bind two common laccase substrates and three industrial dyes. The outcome of this analysis (Figure 8 and Appendix Figure B6) revealed several critical contacts within the substrate‐binding interface. Moreover, some amino acid residues (His527, Trp457, and Thr256) appear to frequently contact the studied substrates.

In summary, the present study demonstrated the capacity of *G. murorum* laccase‐like enzyme to decolorize many industrial dyes and provide several structural insights concerning the putative structure of this enzyme and its interaction with the tested substrates. In addition, we demonstrate the production of a laccase‐like enzyme by *C. thromboides* and reveal its identity. Although this enzyme can process regular laccase substrates (e.g., ABTS and guaiacol), its substrate specificity appears to be different from that of *G. murorum* and merits further analysis. Defining the enzyme−substrate interactions that make some laccases more stable and efficient than others can help to design new enzymes with improved biochemical properties, thus meeting the current needs in laccases suitable for different biotechnological applications.

## AUTHOR CONTRIBUTIONS

**Daniel Fernández‐Remacha**: formal analysis (lead); investigation (lead); writing—original draft (supporting); writing—review and editing (supporting). **Candela González‐Riancho**: formal analysis (equal); investigation (equal); writing—original draft (supporting); writing—review and editing (supporting). **Miranda Lastra Osua**: formal analysis (supporting); investigation (supporting); writing—original draft (supporting); writing—review and editing (supporting). **Aránzazu González Arce**: Formal analysis (supporting); Investigation (supporting); Writing—review and editing (supporting). **Itxaso Montánchez**: funding acquisition (supporting); investigation (supporting); supervision (supporting); writing—review and editing (supporting). **Juan María García‐Lobo**: formal analysis (supporting); investigation (supporting); supervision (supporting); writing—review and editing (supporting). **Roger Estrada‐Tejedor**: formal analysis (supporting); investigation (supporting); methodology (equal); resources (supporting); supervision (equal); writing—original draft (supporting); writing—review and editing (supporting). **Vladimir R. Kaberdin**: conceptualization (equal); formal analysis (equal); funding acquisition (lead); investigation (supporting); project administration (lead); supervision (equal); writing—original draft (lead); writing—review and editing (lead).

## CONFLICTS OF INTEREST

None declared.

## ETHICS STATEMENT

None required.

## Data Availability

All data are provided in full in this paper.

## APPENDIX A

See Tables 1, A1, A2, A3.

**Table A1:** Preliminary genotyping of fungal isolates

Isolate name	Region	Species	Coverage (%)	Identity (%)
H2	*ITS*	*Epicoccum nigrum*	100	100
	*D1/D2*	*Epicoccum nigrum*	99	99
H4	*ITS*	*Epicoccum nigrum*	100	100
	*D1/D2*	*Psiloglonium pusillum*	100	99
H8	*ITS*	*Epicoccum nigrum*	100	100
	*D1/D2*	*Phoma sp.*	100	100
H13	*ITS*	*Gliomastix murorum*	100	98
	*D1/D2*	*Gliomastix murorum*	100	99
H18	*ITS*	*Malassezia restricta*	97	99
	*D1/D2*	*Conidiobolus thromboides*	100	100

*Note*: Assignment to particular species was performed based on the similarity found between their ITS and D1/D2 regions and reference sequences present in the NCBI database. Isolates (H2, H4, and H18) whose ITS and D1/D2 demonstrate the highest homology to phylogenetically distant species could not be assigned unambiguously and therefore were considered for posterior multilocus analysis.

Abbreviation: ITS, Internal Transcribed Spacer.

**Table A2:** Primes used for multilocus sequence analysis of fungal isolates

Primer ID	Sequence (5' → 3')	Orientation	Target gene/region
Fu1‐F Fu1‐R	TCCGTAGGTGAACCTGCGG TCCTCCGCTTATTGATATGC	Forward Reverse	Internal Transcribed Spacer (ITS)
Fu2‐F Fu2‐R	GCATATCAATAAGCGGAGGAAAAG GGTCCGTGTTTCAAGACG	Forward Reverse	D1/D2 in 28S nuclear ribosomal DNA
mtSSU‐F mtSSU‐R	AGTGGGGAATGTTGGGCAAT TCCTCCGCTTATTGATATGC	Forward Reverse	Mitochondrial small subunit rRNA gene
SSU1‐F SSU1‐R (set 1)	AAACGGCTACCACATCCAAG GTTTCAGCCTTGCGACCATA	Forward Reverse	Small subunit rRNA gene
SSU2‐F SSU2‐R (set2)	TAACTTCTGCGAAAGCATTT TCCTTTTCGCTACCCACACT	Forward Reverse	Small subunit rRNA gene
TEF1‐F TEF1‐R	ATGGCCAGACTCGTGAACAC ACGATGACCTGGGCGTTGAA	Forward Reverse	Translation elongation factor EF‐1 alpha
RPB2‐F RPB2‐R	CCGTACTTAYWTGGAYCARG GTCATWCGDGADGGDATDGC	Forward Reverse	DNA‐directed RNA polymerase II core subunit
TUB2‐F	TGCTTTCTGGCAGACCATCT	Forward	Beta‐tubulin
TUB2‐R	CCCTTGGCCCAGTTGTTACC	Reverse	

**Table A3:** Sequence similarity of the putative *G. murorum* oxidase and laccase‐like enzymes with available crystallographic structures

Organism	Protein (ID of protein structure)	*G. murorum*	*A. verrucaria*	*M. albomyces*	*T. versicolor*
*G. murorum*	Laccase	—	65.1%	16.6%	18.0%
*A. verrucaria*	Bilirubin oxidase (6IQZ)		—	15.6%	17.6%
*M. albomyces*	Laccase (1GW0)			—	28.5%
*T. versicolor*	Laccase (1GYC)				—

## APPENDIX B

See Figures B1, B2, B3, B4, B5, B6.
